# Chlorido[2,2′-(oxydimethyl­ene)­dipyridine]copper(II) perchlorate–aqua­chlorido[2,2′-(oxydimethyl­ene)­dipyridine]copper(II) perchlorate (1/1)

**DOI:** 10.1107/S1600536809027123

**Published:** 2009-07-18

**Authors:** Hong Li, Long Miao Xie, Shi Guo Zhang

**Affiliations:** a Department of Chemistry and Chemical Engineering, Institute of Materials Chemistry, Binzhou University, Binzhou 256603, People’s Republic of China; b Department of Chemistry, Shandong Normal University, Jinan 250014, People’s Republic of China

## Abstract

The asymmetric unit of the title compound, [CuCl(C_12_H_12_N_2_O)][CuCl(C_12_H_12_N_2_O)(H_2_O)](ClO_4_)_2_, contains two different discrete cations. In one cation, the Cu^II^ ion is coordinated in a slightly distorted square-planar geometry, while in the other the Cu^II^ ion is in a slightly distorted square-pyramidal environment. In the crystal structure, there are O—H⋯O hydrogen bonds between coordinated water mol­ecules and perchlorate anions. Both types of cations are linked into one-dimensional chains along the *b* axis by weak electrostatic Cu⋯Cl inter­actions, with Cu⋯Cl distances of 2.8088 (16) and 3.2074 (17) Å.

## Related literature

For related structures, see: Li (2007 ▶, 2008*a* ▶,*b* ▶).
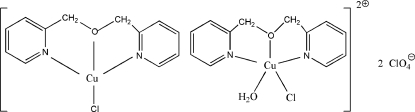


## Experimental

### 

#### Crystal data

[CuCl(C_12_H_12_N_2_O)][CuCl(C_12_H_12_N_2_O)(H_2_O)](ClO_4_)_2_
*M* *_r_* = 815.37
Triclinic, 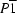

*a* = 10.997 (2) Å
*b* = 12.882 (3) Å
*c* = 12.913 (3) Å
α = 97.174 (3)°
β = 112.031 (3)°
γ = 106.851 (3)°
*V* = 1565.7 (5) Å^3^
*Z* = 2
Mo *K*α radiation
μ = 1.76 mm^−1^
*T* = 298 K
0.42 × 0.23 × 0.21 mm

#### Data collection

Bruker SMART APEX CCD diffractometer
Absorption correction: multi-scan (*SADABS*; Sheldrick, 1996 ▶) *T*_min_ = 0.525, *T*_max_ = 0.709
8530 measured reflections
6009 independent reflections
4063 reflections with *I* > 2σ(*I*)
*R*_int_ = 0.024

#### Refinement

*R*[*F* ^2^ > 2σ(*F* ^2^)] = 0.062
*wR*(*F* ^2^) = 0.185
*S* = 1.05
6009 reflections
411 parameters
3 restraints
H-atom parameters constrained
Δρ_max_ = 1.01 e Å^−3^
Δρ_min_ = −0.60 e Å^−3^

### 

Data collection: *SMART* (Bruker, 1997 ▶); cell refinement: *SAINT* (Bruker, 1997 ▶); data reduction: *SAINT*; program(s) used to solve structure: *SHELXTL* (Sheldrick, 2008 ▶); program(s) used to refine structure: *SHELXTL*; molecular graphics: *SHELXTL* and *PLATON* (Spek, 2009 ▶); software used to prepare material for publication: *SHELXTL*.

## Supplementary Material

Crystal structure: contains datablocks I, global. DOI: 10.1107/S1600536809027123/lh2860sup1.cif

Structure factors: contains datablocks I. DOI: 10.1107/S1600536809027123/lh2860Isup2.hkl

Additional supplementary materials:  crystallographic information; 3D view; checkCIF report

## Figures and Tables

**Table:** 

Cl3—Cu2	2.2511 (15)
Cl4—Cu1	2.2067 (14)
Cu1—N3	1.968 (4)
Cu1—O1	1.970 (3)
Cu1—N4	1.973 (4)
Cu2—N2	1.970 (4)
Cu2—N1	1.972 (4)
Cu2—O2	2.005 (4)
Cu2—O11	2.298 (4)

**Table:** 

N3—Cu1—O1	80.97 (17)
N3—Cu1—N4	161.79 (19)
O1—Cu1—N4	81.04 (17)
N3—Cu1—Cl4	98.72 (13)
O1—Cu1—Cl4	173.09 (12)
N4—Cu1—Cl4	98.80 (14)
N2—Cu2—N1	159.85 (19)
N2—Cu2—O2	80.78 (17)
N1—Cu2—O2	80.45 (16)
N2—Cu2—Cl3	98.32 (14)
N1—Cu2—Cl3	98.09 (13)
O2—Cu2—Cl3	165.86 (13)
N2—Cu2—O11	93.89 (17)
N1—Cu2—O11	92.93 (17)
O2—Cu2—O11	88.68 (16)
Cl3—Cu2—O11	105.46 (12)

**Table 2:** Hydrogen-bond geometry (Å, °)

*D*—H⋯*A*	*D*—H	H⋯*A*	*D*⋯*A*	*D*—H⋯*A*
O11—H11*A*⋯O5^i^	0.90	2.05	2.725 (8)	131
O11—H11*B*⋯O9^ii^	0.90	1.92	2.787 (10)	163

Symmetry codes: (i) 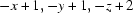; (ii) 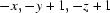.

**Fig. 1.**
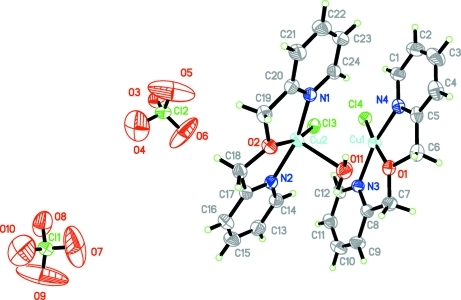
The asymmetric unit of the title complex with the atom-numbering scheme. Displacement ellipsoids are drawn at the 30% probability level.

**Fig. 2.**
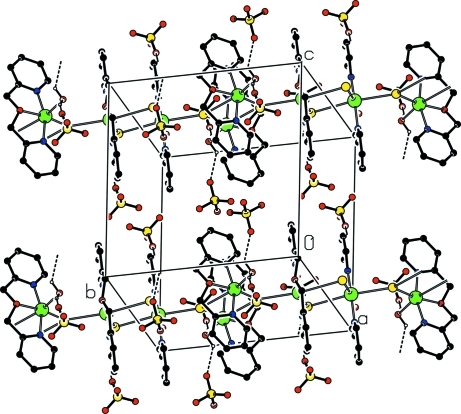
Part of the crystal structure with hydrogen bonds shown as dashed lines.

## supplementary crystallographic information

### Comment

Derivatives of pyridine play an important role in modern coordination chemistry and some complexes using 2,2'-(oxydimethylene)dipyridine as a ligand have already been reported (Li, 2007, 2008*a*,*b*). Herein we report the crystal structure of the title compound.

The molecular structure of the title compound is shown in Fig. 1. Atom Cu1 is in a slightly distorted square-planar coordination environment and atom Cu2 is coordinated in a slightly distorted square-pyramidal environment with the O atom of the coordinated H_2_O ligand in the apical site. 2,2'-(oxydimethylene)dipyridine acts as a tridentate ligand as in the related Cu^II^, Zn^II^ and Cd^II^ complexes (Li, 2007, 2008a,b). In the crystal structure, there are O—H···O hydrogen bonds between coordinated water molecule and perchlorate ions and both types of cation are linked into one-dimensional chains along the b axis by weak electrostatic Cu···Cl interactions with Cu···Cl distances of 2.8088 (16) and 3.2074 (17) Å (see Fig. 2).

### Experimental

An 8ml methanol solution of 2,2'-(oxydimetheylene)dipyridine (0.0386 g, 0.193 mmol) was added to an 8 ml H_2_O solution containing Cu(ClO_4_)_2_.6H_2_O (0.0730 g, 0.197 mmol), and the mixture was stirred for a few minutes. Then, diluted HCl solution was added into the mixed solution in drops until the pH = 4.0. Blue single crystals were obtained after the solution had been allowed to stand at room temperature for three weeks.

### Refinement

H_2_O-bound H atoms were located in a difference Fourier map, and placed in idealized positions with O—H = 0.90 Å and with *U*_iso_(H) = 1.5*U*_eq_(O); other H atoms were placed in calculated positions with C—H = 0.97 Å for methylene group and C—H = 0.93 Å for pyridyl group with *U*_iso_(H) = 1.2*U*_eq_(C). All H atoms were refined in a riding-model approximation.

### Crystal data

**Table:**

[CuCl(C_12_H_12_N_2_O)][CuCl(C_12_H_12_N_2_O)(H_2_O)](ClO_4_)_2_	*Z* = 2
*M**_r_* = 815.37	*F*(000) = 824
Triclinic, *P*1	*D*_x_ = 1.730 Mg m^−^^3^
Hall symbol: -P 1	Mo *K*α radiation, λ = 0.71073 Å
*a* = 10.997 (2) Å	Cell parameters from 2082 reflections
*b* = 12.882 (3) Å	θ = 2.2–23.8°
*c* = 12.913 (3) Å	µ = 1.76 mm^−^^1^
α = 97.174 (3)°	*T* = 298 K
β = 112.031 (3)°	Block, blue
γ = 106.851 (3)°	0.42 × 0.23 × 0.21 mm
*V* = 1565.7 (5) Å^3^	

### Data collection

**Table:**

Bruker SMART APEX CCD diffractometer	6009 independent reflections
Radiation source: fine-focus sealed tube	4063 reflections with *I* > 2σ(*I*)
graphite	*R*_int_ = 0.024
φ and ω scans	θ_max_ = 26.0°, θ_min_ = 1.7°
Absorption correction: multi-scan (*SADABS*; Sheldrick, 1996)	*h* = −13→10
*T*_min_ = 0.525, *T*_max_ = 0.709	*k* = −15→15
8530 measured reflections	*l* = −15→15

### Refinement

**Table:**

Refinement on *F*^2^	Primary atom site location: structure-invariant direct methods
Least-squares matrix: full	Secondary atom site location: difference Fourier map
*R*[*F*^2^ > 2σ(*F*^2^)] = 0.062	Hydrogen site location: inferred from neighbouring sites
*wR*(*F*^2^) = 0.185	H-atom parameters constrained
*S* = 1.05	*w* = 1/[σ^2^(*F*_o_^2^) + (0.1088*P*)^2^] where *P* = (*F*_o_^2^ + 2*F*_c_^2^)/3
6009 reflections	(Δ/σ)_max_ < 0.001
411 parameters	Δρ_max_ = 1.01 e Å^−^^3^
3 restraints	Δρ_min_ = −0.60 e Å^−^^3^

### Special details

**Table:**

Geometry. All e.s.d.'s (except the e.s.d. in the dihedral angle between two l.s. planes) are estimated using the full covariance matrix. The cell e.s.d.'s are taken into account individually in the estimation of e.s.d.'s in distances, angles and torsion angles; correlations between e.s.d.'s in cell parameters are only used when they are defined by crystal symmetry. An approximate (isotropic) treatment of cell e.s.d.'s is used for estimating e.s.d.'s involving l.s. planes.
Refinement. Refinement of *F*^2^ against ALL reflections. The weighted *R*-factor *wR* and goodness of fit *S* are based on *F*^2^, conventional *R*-factors *R* are based on *F*, with *F* set to zero for negative *F*^2^. The threshold expression of *F*^2^ > σ(*F*^2^) is used only for calculating *R*-factors(gt) *etc*. and is not relevant to the choice of reflections for refinement. *R*-factors based on *F*^2^ are statistically about twice as large as those based on *F*, and *R*- factors based on ALL data will be even larger.

### Fractional atomic coordinates and isotropic or equivalent isotropic displacement parameters (Å^2^)

**Table:**

	*x*	*y*	*z*	*U*_iso_*/*U*_eq_	
C1	0.6320 (7)	0.2031 (5)	1.2505 (5)	0.0595 (16)	
H1	0.7080	0.2218	1.2317	0.071*	
C2	0.6551 (8)	0.2240 (6)	1.3637 (6)	0.078 (2)	
H2	0.7464	0.2573	1.4224	0.093*	
C3	0.5399 (10)	0.1947 (7)	1.3902 (6)	0.085 (2)	
H3	0.5543	0.2069	1.4671	0.102*	
C4	0.4070 (9)	0.1483 (6)	1.3042 (6)	0.073 (2)	
H4	0.3296	0.1303	1.3211	0.088*	
C5	0.3891 (7)	0.1283 (5)	1.1908 (5)	0.0507 (14)	
C6	0.2451 (6)	0.0723 (5)	1.0915 (5)	0.0567 (15)	
H6A	0.2091	−0.0075	1.0855	0.068*	
H6B	0.1803	0.1047	1.1031	0.068*	
C7	0.1462 (6)	0.0206 (5)	0.8805 (5)	0.0583 (15)	
H7A	0.0685	0.0471	0.8607	0.070*	
H7B	0.1126	−0.0563	0.8852	0.070*	
C8	0.2003 (6)	0.0257 (5)	0.7912 (5)	0.0499 (13)	
C9	0.1103 (7)	−0.0147 (6)	0.6740 (6)	0.0661 (17)	
H9	0.0131	−0.0438	0.6492	0.079*	
C10	0.1668 (8)	−0.0112 (7)	0.5942 (6)	0.074 (2)	
H10	0.1084	−0.0395	0.5153	0.089*	
C11	0.3108 (8)	0.0351 (6)	0.6341 (5)	0.0664 (17)	
H11	0.3509	0.0395	0.5821	0.080*	
C12	0.3942 (6)	0.0742 (5)	0.7500 (5)	0.0514 (14)	
H12	0.4914	0.1050	0.7763	0.062*	
C13	0.1523 (7)	0.3151 (6)	0.6299 (5)	0.0659 (17)	
H13	0.1544	0.2623	0.5751	0.079*	
C14	0.2190 (6)	0.3236 (5)	0.7442 (5)	0.0572 (15)	
H14	0.2672	0.2757	0.7668	0.069*	
C15	0.0808 (7)	0.3866 (6)	0.5959 (5)	0.073 (2)	
H15	0.0355	0.3836	0.5180	0.088*	
C16	0.0780 (7)	0.4616 (6)	0.6790 (5)	0.0682 (18)	
H16	0.0288	0.5091	0.6576	0.082*	
C17	0.1485 (6)	0.4672 (5)	0.7953 (5)	0.0500 (13)	
C18	0.1488 (7)	0.5490 (5)	0.8875 (5)	0.0558 (14)	
H18A	0.2063	0.6247	0.8940	0.067*	
H18B	0.0536	0.5460	0.8689	0.067*	
C19	0.2582 (7)	0.6040 (5)	1.0978 (5)	0.0579 (15)	
H19A	0.1815	0.6096	1.1152	0.069*	
H19B	0.3072	0.6762	1.0898	0.069*	
C20	0.3576 (6)	0.5727 (5)	1.1929 (5)	0.0489 (13)	
C21	0.4126 (7)	0.6304 (6)	1.3080 (5)	0.0646 (17)	
H21	0.3854	0.6890	1.3279	0.077*	
C22	0.5060 (7)	0.6021 (6)	1.3923 (6)	0.0704 (18)	
H22	0.5467	0.6431	1.4693	0.084*	
C23	0.5389 (7)	0.5114 (6)	1.3611 (5)	0.0645 (17)	
H23	0.5995	0.4882	1.4172	0.077*	
C24	0.4815 (6)	0.4558 (5)	1.2464 (5)	0.0555 (15)	
H24	0.5037	0.3944	1.2257	0.067*	
Cl1	0.19499 (15)	0.84371 (14)	0.29265 (13)	0.0570 (4)	
Cl2	0.89001 (17)	0.69411 (16)	0.70403 (14)	0.0648 (4)	
Cl3	0.47655 (15)	0.34451 (11)	0.99732 (12)	0.0504 (4)	
Cl4	0.65816 (14)	0.13410 (12)	1.00771 (12)	0.0496 (3)	
Cu1	0.44927 (6)	0.12094 (6)	0.99775 (5)	0.0443 (2)	
Cu2	0.31420 (7)	0.41888 (6)	0.99518 (5)	0.0482 (2)	
N1	0.3943 (5)	0.4871 (4)	1.1630 (4)	0.0453 (10)	
N2	0.2185 (5)	0.3987 (4)	0.8269 (4)	0.0483 (11)	
N3	0.3399 (5)	0.0696 (4)	0.8284 (4)	0.0455 (10)	
N4	0.4983 (5)	0.1550 (4)	1.1649 (4)	0.0492 (11)	
O1	0.2589 (4)	0.0900 (3)	0.9887 (3)	0.0487 (9)	
O2	0.2049 (4)	0.5195 (3)	0.9940 (3)	0.0533 (10)	
O3	1.0211 (5)	0.7169 (5)	0.6997 (5)	0.0934 (16)	
O4	0.8206 (10)	0.7530 (10)	0.6406 (10)	0.221 (5)	
O5	0.9105 (10)	0.7257 (15)	0.8086 (7)	0.335 (10)	
O6	0.8156 (9)	0.5888 (7)	0.6593 (12)	0.253 (7)	
O7	0.1715 (12)	0.7738 (12)	0.3473 (14)	0.304 (9)	
O8	0.3380 (7)	0.8819 (6)	0.3202 (8)	0.157 (3)	
O9	0.1338 (12)	0.7967 (16)	0.1870 (8)	0.356 (12)	
O10	0.1404 (10)	0.9200 (9)	0.3042 (11)	0.218 (5)	
O11	0.1331 (5)	0.2760 (4)	0.9973 (4)	0.0675 (11)	
H11A	0.1292	0.3169	1.0566	0.101*	
H11B	0.0516	0.2676	0.9381	0.101*	

### Atomic displacement parameters (Å^2^)

**Table:**

	*U*^11^	*U*^22^	*U*^33^	*U*^12^	*U*^13^	*U*^23^
C1	0.065 (4)	0.061 (4)	0.047 (3)	0.026 (3)	0.018 (3)	0.009 (3)
C2	0.082 (5)	0.078 (5)	0.053 (4)	0.030 (4)	0.012 (4)	0.004 (3)
C3	0.125 (7)	0.099 (6)	0.048 (4)	0.060 (6)	0.042 (5)	0.017 (4)
C4	0.102 (6)	0.080 (5)	0.072 (5)	0.050 (5)	0.057 (5)	0.031 (4)
C5	0.071 (4)	0.045 (3)	0.057 (3)	0.033 (3)	0.039 (3)	0.019 (3)
C6	0.065 (4)	0.056 (4)	0.072 (4)	0.030 (3)	0.046 (3)	0.023 (3)
C7	0.042 (3)	0.061 (4)	0.070 (4)	0.021 (3)	0.019 (3)	0.018 (3)
C8	0.047 (3)	0.048 (3)	0.056 (3)	0.024 (3)	0.018 (3)	0.018 (3)
C9	0.045 (3)	0.071 (4)	0.064 (4)	0.023 (3)	0.005 (3)	0.016 (3)
C10	0.077 (5)	0.088 (5)	0.049 (4)	0.039 (4)	0.012 (3)	0.017 (4)
C11	0.083 (5)	0.072 (5)	0.052 (4)	0.033 (4)	0.032 (3)	0.018 (3)
C12	0.054 (3)	0.060 (4)	0.046 (3)	0.024 (3)	0.024 (3)	0.020 (3)
C13	0.079 (5)	0.060 (4)	0.053 (4)	0.015 (4)	0.033 (3)	0.008 (3)
C14	0.066 (4)	0.052 (4)	0.047 (3)	0.016 (3)	0.024 (3)	0.008 (3)
C15	0.078 (5)	0.085 (5)	0.041 (4)	0.016 (4)	0.021 (3)	0.014 (3)
C16	0.073 (4)	0.078 (5)	0.048 (4)	0.030 (4)	0.017 (3)	0.025 (3)
C17	0.047 (3)	0.052 (4)	0.047 (3)	0.013 (3)	0.020 (3)	0.017 (3)
C18	0.061 (4)	0.054 (4)	0.054 (3)	0.027 (3)	0.022 (3)	0.018 (3)
C19	0.080 (4)	0.053 (4)	0.056 (4)	0.037 (3)	0.037 (3)	0.015 (3)
C20	0.055 (3)	0.046 (3)	0.050 (3)	0.017 (3)	0.029 (3)	0.009 (3)
C21	0.086 (5)	0.062 (4)	0.049 (4)	0.032 (4)	0.031 (3)	0.009 (3)
C22	0.081 (5)	0.076 (5)	0.049 (4)	0.022 (4)	0.032 (3)	0.002 (3)
C23	0.063 (4)	0.072 (5)	0.046 (3)	0.021 (3)	0.013 (3)	0.016 (3)
C24	0.070 (4)	0.048 (3)	0.051 (3)	0.023 (3)	0.027 (3)	0.016 (3)
Cl1	0.0530 (9)	0.0720 (11)	0.0519 (9)	0.0266 (8)	0.0252 (7)	0.0185 (8)
Cl2	0.0521 (9)	0.0780 (12)	0.0550 (9)	0.0191 (8)	0.0207 (7)	0.0063 (8)
Cl3	0.0574 (8)	0.0420 (8)	0.0598 (8)	0.0232 (6)	0.0293 (7)	0.0150 (6)
Cl4	0.0424 (7)	0.0506 (8)	0.0599 (8)	0.0209 (6)	0.0233 (6)	0.0149 (6)
Cu1	0.0410 (4)	0.0507 (4)	0.0428 (4)	0.0176 (3)	0.0196 (3)	0.0103 (3)
Cu2	0.0602 (5)	0.0470 (4)	0.0409 (4)	0.0263 (3)	0.0209 (3)	0.0102 (3)
N1	0.054 (3)	0.041 (3)	0.041 (2)	0.016 (2)	0.021 (2)	0.010 (2)
N2	0.057 (3)	0.041 (3)	0.043 (3)	0.014 (2)	0.021 (2)	0.010 (2)
N3	0.046 (3)	0.045 (3)	0.043 (3)	0.017 (2)	0.017 (2)	0.010 (2)
N4	0.063 (3)	0.048 (3)	0.047 (3)	0.027 (2)	0.030 (2)	0.016 (2)
O1	0.049 (2)	0.051 (2)	0.057 (2)	0.0208 (18)	0.0314 (19)	0.0182 (19)
O2	0.066 (3)	0.054 (2)	0.045 (2)	0.033 (2)	0.0210 (18)	0.0109 (18)
O3	0.061 (3)	0.107 (4)	0.113 (4)	0.026 (3)	0.042 (3)	0.029 (3)
O4	0.147 (8)	0.260 (12)	0.351 (15)	0.151 (9)	0.128 (9)	0.141 (11)
O5	0.136 (8)	0.70 (3)	0.089 (6)	0.059 (12)	0.076 (6)	−0.015 (10)
O6	0.111 (6)	0.093 (6)	0.52 (2)	−0.001 (5)	0.152 (10)	0.014 (9)
O7	0.200 (10)	0.398 (19)	0.52 (2)	0.167 (12)	0.248 (14)	0.384 (19)
O8	0.078 (4)	0.124 (6)	0.285 (10)	0.039 (4)	0.084 (5)	0.091 (6)
O9	0.201 (11)	0.66 (3)	0.104 (7)	0.225 (16)	−0.020 (7)	−0.122 (12)
O10	0.153 (7)	0.171 (9)	0.366 (14)	0.118 (7)	0.112 (8)	0.048 (9)
O11	0.071 (3)	0.066 (3)	0.066 (3)	0.019 (2)	0.035 (2)	0.019 (2)

### Geometric parameters (Å, °)

**Table:**

C1—N4	1.360 (7)	C17—C18	1.486 (8)
C1—C2	1.364 (8)	C18—O2	1.430 (6)
C1—H1	0.9300	C18—H18A	0.9700
C2—C3	1.394 (11)	C18—H18B	0.9700
C2—H2	0.9300	C19—O2	1.415 (6)
C3—C4	1.356 (10)	C19—C20	1.496 (8)
C3—H3	0.9300	C19—H19A	0.9700
C4—C5	1.381 (8)	C19—H19B	0.9700
C4—H4	0.9300	C20—N1	1.340 (7)
C5—N4	1.328 (7)	C20—C21	1.385 (8)
C5—C6	1.502 (9)	C21—C22	1.360 (9)
C6—O1	1.430 (6)	C21—H21	0.9300
C6—H6A	0.9700	C22—C23	1.377 (9)
C6—H6B	0.9700	C22—H22	0.9300
C7—O1	1.426 (7)	C23—C24	1.370 (8)
C7—C8	1.483 (8)	C23—H23	0.9300
C7—H7A	0.9700	C24—N1	1.342 (7)
C7—H7B	0.9700	C24—H24	0.9300
C8—N3	1.335 (7)	Cl1—O7	1.239 (8)
C8—C9	1.386 (8)	Cl1—O9	1.244 (8)
C9—C10	1.388 (10)	Cl1—O10	1.313 (7)
C9—H9	0.9300	Cl1—O8	1.389 (6)
C10—C11	1.376 (9)	Cl2—O5	1.273 (7)
C10—H10	0.9300	Cl2—O6	1.285 (8)
C11—C12	1.360 (8)	Cl2—O4	1.360 (8)
C11—H11	0.9300	Cl2—O3	1.409 (5)
C12—N3	1.353 (7)	Cl3—Cu2	2.2511 (15)
C12—H12	0.9300	Cl4—Cu1	2.2067 (14)
C13—C14	1.353 (8)	Cu1—N3	1.968 (4)
C13—C15	1.386 (9)	Cu1—O1	1.970 (3)
C13—H13	0.9300	Cu1—N4	1.973 (4)
C14—N2	1.352 (7)	Cu2—N2	1.970 (4)
C14—H14	0.9300	Cu2—N1	1.972 (4)
C15—C16	1.367 (9)	Cu2—O2	2.005 (4)
C15—H15	0.9300	Cu2—O11	2.298 (4)
C16—C17	1.389 (8)	O11—H11A	0.8969
C16—H16	0.9300	O11—H11B	0.8995
C17—N2	1.336 (7)		
			
N4—C1—C2	120.2 (6)	C20—C19—H19B	110.1
N4—C1—H1	119.9	H19A—C19—H19B	108.4
C2—C1—H1	119.9	N1—C20—C21	120.4 (5)
C1—C2—C3	119.1 (7)	N1—C20—C19	117.7 (5)
C1—C2—H2	120.5	C21—C20—C19	121.9 (5)
C3—C2—H2	120.5	C22—C21—C20	120.6 (6)
C4—C3—C2	120.2 (6)	C22—C21—H21	119.7
C4—C3—H3	119.9	C20—C21—H21	119.7
C2—C3—H3	119.9	C21—C22—C23	118.5 (6)
C3—C4—C5	118.6 (7)	C21—C22—H22	120.8
C3—C4—H4	120.7	C23—C22—H22	120.8
C5—C4—H4	120.7	C24—C23—C22	119.2 (6)
N4—C5—C4	121.6 (6)	C24—C23—H23	120.4
N4—C5—C6	117.1 (5)	C22—C23—H23	120.4
C4—C5—C6	121.3 (6)	N1—C24—C23	122.1 (6)
O1—C6—C5	107.2 (5)	N1—C24—H24	119.0
O1—C6—H6A	110.3	C23—C24—H24	119.0
C5—C6—H6A	110.3	O7—Cl1—O9	109.6 (11)
O1—C6—H6B	110.3	O7—Cl1—O10	111.8 (7)
C5—C6—H6B	110.3	O9—Cl1—O10	104.2 (8)
H6A—C6—H6B	108.5	O7—Cl1—O8	108.2 (6)
O1—C7—C8	108.0 (5)	O9—Cl1—O8	105.6 (7)
O1—C7—H7A	110.1	O10—Cl1—O8	117.1 (6)
C8—C7—H7A	110.1	O5—Cl2—O6	111.9 (9)
O1—C7—H7B	110.1	O5—Cl2—O4	108.8 (9)
C8—C7—H7B	110.1	O6—Cl2—O4	108.4 (7)
H7A—C7—H7B	108.4	O5—Cl2—O3	109.3 (5)
N3—C8—C9	121.3 (5)	O6—Cl2—O3	109.4 (5)
N3—C8—C7	117.1 (5)	O4—Cl2—O3	108.9 (5)
C9—C8—C7	121.6 (6)	N3—Cu1—O1	80.97 (17)
C8—C9—C10	119.2 (6)	N3—Cu1—N4	161.79 (19)
C8—C9—H9	120.4	O1—Cu1—N4	81.04 (17)
C10—C9—H9	120.4	N3—Cu1—Cl4	98.72 (13)
C11—C10—C9	118.8 (6)	O1—Cu1—Cl4	173.09 (12)
C11—C10—H10	120.6	N4—Cu1—Cl4	98.80 (14)
C9—C10—H10	120.6	N2—Cu2—N1	159.85 (19)
C12—C11—C10	119.5 (6)	N2—Cu2—O2	80.78 (17)
C12—C11—H11	120.2	N1—Cu2—O2	80.45 (16)
C10—C11—H11	120.2	N2—Cu2—Cl3	98.32 (14)
N3—C12—C11	122.0 (6)	N1—Cu2—Cl3	98.09 (13)
N3—C12—H12	119.0	O2—Cu2—Cl3	165.86 (13)
C11—C12—H12	119.0	N2—Cu2—O11	93.89 (17)
C14—C13—C15	118.8 (6)	N1—Cu2—O11	92.93 (17)
C14—C13—H13	120.6	O2—Cu2—O11	88.68 (16)
C15—C13—H13	120.6	Cl3—Cu2—O11	105.46 (12)
N2—C14—C13	122.7 (6)	C20—N1—C24	119.1 (5)
N2—C14—H14	118.6	C20—N1—Cu2	115.3 (4)
C13—C14—H14	118.6	C24—N1—Cu2	125.5 (4)
C16—C15—C13	118.8 (6)	C17—N2—C14	119.1 (5)
C16—C15—H15	120.6	C17—N2—Cu2	115.1 (4)
C13—C15—H15	120.6	C14—N2—Cu2	125.8 (4)
C15—C16—C17	120.2 (6)	C8—N3—C12	119.2 (5)
C15—C16—H16	119.9	C8—N3—Cu1	115.0 (4)
C17—C16—H16	119.9	C12—N3—Cu1	125.8 (4)
N2—C17—C16	120.4 (6)	C5—N4—C1	120.3 (5)
N2—C17—C18	118.3 (5)	C5—N4—Cu1	115.0 (4)
C16—C17—C18	121.3 (6)	C1—N4—Cu1	124.6 (4)
O2—C18—C17	107.8 (5)	C7—O1—C6	117.4 (5)
O2—C18—H18A	110.2	C7—O1—Cu1	115.1 (3)
C17—C18—H18A	110.2	C6—O1—Cu1	115.1 (3)
O2—C18—H18B	110.2	C19—O2—C18	117.2 (4)
C17—C18—H18B	110.2	C19—O2—Cu2	115.9 (3)
H18A—C18—H18B	108.5	C18—O2—Cu2	115.2 (3)
O2—C19—C20	107.9 (4)	Cu2—O11—H11A	94.6
O2—C19—H19A	110.1	Cu2—O11—H11B	109.4
C20—C19—H19A	110.1	H11A—O11—H11B	101.6
O2—C19—H19B	110.1		
			
N4—C1—C2—C3	0.5 (10)	O11—Cu2—N2—C17	96.4 (4)
C1—C2—C3—C4	−1.4 (11)	N1—Cu2—N2—C14	166.6 (5)
C2—C3—C4—C5	1.7 (11)	O2—Cu2—N2—C14	−171.9 (5)
C3—C4—C5—N4	−1.1 (10)	Cl3—Cu2—N2—C14	22.4 (5)
C3—C4—C5—C6	177.2 (6)	O11—Cu2—N2—C14	−83.8 (5)
N4—C5—C6—O1	−16.3 (7)	C9—C8—N3—C12	−0.3 (8)
C4—C5—C6—O1	165.4 (5)	C7—C8—N3—C12	178.8 (5)
O1—C7—C8—N3	13.7 (7)	C9—C8—N3—Cu1	−179.5 (4)
O1—C7—C8—C9	−167.1 (5)	C7—C8—N3—Cu1	−0.3 (6)
N3—C8—C9—C10	1.2 (9)	C11—C12—N3—C8	−0.2 (8)
C7—C8—C9—C10	−177.9 (6)	C11—C12—N3—Cu1	178.9 (4)
C8—C9—C10—C11	−1.6 (10)	O1—Cu1—N3—C8	−9.1 (4)
C9—C10—C11—C12	1.2 (10)	N4—Cu1—N3—C8	−0.1 (8)
C10—C11—C12—N3	−0.3 (10)	Cl4—Cu1—N3—C8	163.9 (4)
C15—C13—C14—N2	0.2 (10)	O1—Cu1—N3—C12	171.7 (5)
C14—C13—C15—C16	−1.2 (10)	N4—Cu1—N3—C12	−179.2 (5)
C13—C15—C16—C17	1.4 (10)	Cl4—Cu1—N3—C12	−15.2 (5)
C15—C16—C17—N2	−0.6 (9)	C4—C5—N4—C1	0.1 (8)
C15—C16—C17—C18	179.2 (6)	C6—C5—N4—C1	−178.2 (5)
N2—C17—C18—O2	−11.5 (7)	C4—C5—N4—Cu1	−179.3 (5)
C16—C17—C18—O2	168.8 (5)	C6—C5—N4—Cu1	2.3 (6)
O2—C19—C20—N1	10.1 (7)	C2—C1—N4—C5	0.1 (9)
O2—C19—C20—C21	−171.0 (5)	C2—C1—N4—Cu1	179.6 (5)
N1—C20—C21—C22	0.8 (9)	N3—Cu1—N4—C5	−0.5 (8)
C19—C20—C21—C22	−178.0 (6)	O1—Cu1—N4—C5	8.5 (4)
C20—C21—C22—C23	−3.2 (10)	Cl4—Cu1—N4—C5	−164.5 (4)
C21—C22—C23—C24	2.7 (10)	N3—Cu1—N4—C1	−180.0 (5)
C22—C23—C24—N1	0.3 (10)	O1—Cu1—N4—C1	−170.9 (5)
C21—C20—N1—C24	2.1 (8)	Cl4—Cu1—N4—C1	16.1 (5)
C19—C20—N1—C24	−179.0 (5)	C8—C7—O1—C6	−161.6 (4)
C21—C20—N1—Cu2	−177.1 (4)	C8—C7—O1—Cu1	−21.1 (6)
C19—C20—N1—Cu2	1.8 (6)	C5—C6—O1—C7	163.5 (4)
C23—C24—N1—C20	−2.7 (8)	C5—C6—O1—Cu1	23.1 (5)
C23—C24—N1—Cu2	176.4 (4)	N3—Cu1—O1—C7	17.4 (4)
N2—Cu2—N1—C20	12.5 (8)	N4—Cu1—O1—C7	−159.8 (4)
O2—Cu2—N1—C20	−9.0 (4)	Cl4—Cu1—O1—C7	−70.6 (10)
Cl3—Cu2—N1—C20	156.7 (4)	N3—Cu1—O1—C6	158.8 (4)
O11—Cu2—N1—C20	−97.2 (4)	N4—Cu1—O1—C6	−18.4 (4)
N2—Cu2—N1—C24	−166.6 (5)	Cl4—Cu1—O1—C6	70.8 (11)
O2—Cu2—N1—C24	171.8 (5)	C20—C19—O2—C18	−158.7 (5)
Cl3—Cu2—N1—C24	−22.4 (5)	C20—C19—O2—Cu2	−17.4 (6)
O11—Cu2—N1—C24	83.7 (5)	C17—C18—O2—C19	159.6 (5)
C16—C17—N2—C14	−0.5 (8)	C17—C18—O2—Cu2	18.0 (6)
C18—C17—N2—C14	179.8 (5)	N2—Cu2—O2—C19	−157.4 (4)
C16—C17—N2—Cu2	179.3 (4)	N1—Cu2—O2—C19	15.3 (4)
C18—C17—N2—Cu2	−0.4 (7)	Cl3—Cu2—O2—C19	−69.9 (6)
C13—C14—N2—C17	0.6 (9)	O11—Cu2—O2—C19	108.5 (4)
C13—C14—N2—Cu2	−179.1 (5)	N2—Cu2—O2—C18	−15.2 (4)
N1—Cu2—N2—C17	−13.2 (8)	N1—Cu2—O2—C18	157.4 (4)
O2—Cu2—N2—C17	8.4 (4)	Cl3—Cu2—O2—C18	72.2 (6)
Cl3—Cu2—N2—C17	−157.4 (4)	O11—Cu2—O2—C18	−109.4 (4)

### Hydrogen-bond geometry (Å, °)

**Table:**

*D*—H···*A*	*D*—H	H···*A*	*D*···*A*	*D*—H···*A*
O11—H11A···O5^i^	0.90	2.05	2.725 (8)	131
O11—H11B···O9^ii^	0.90	1.92	2.787 (10)	163

Symmetry codes: (i) −*x*+1, −*y*+1, −*z*+2; (ii) −*x*, −*y*+1, −*z*+1.
